# Burnout and patient safety culture assessment in a secondary care hospital

**DOI:** 10.12669/pjms.40.2(ICON).8970

**Published:** 2024-01

**Authors:** Sharmeen Ziarukh, Aamina Sabir

**Affiliations:** 1 Sharmeen Ziarukh, Department of Family Medicine and Nutrition, Managed by IHHN, THQ Manawan Hospital, Lahore, Pakistan; 2 Aamina Sabir, Department of Family Medicine and Nutrition, Managed by IHHN, THQ Manawan Hospital, Lahore, Pakistan

**Keywords:** Burnout, Healthcare System, Healthcare Providers, Patient Safety

## Abstract

**Objectives:**

To identify employee burn-out and assess its impact on patient safety culture.

**Methods:**

This cross-sectional study was carried out amongst healthcare providers (HCP) of Tehsil Head Quarter Manawan Hospital, Lahore from April 1^st^ till 30^th^, 2023, who had been working for at least one year and directly involved in patient care. Two questionnaires were used; the Maslach Burnout Inventory (MBI) to assess level of burnout, and Agency for Healthcare Research and Quality (AHRQ) patient safety culture survey. After obtaining informed consent, 59 participants were enrolled in this study.

**Results:**

High degree of occupational exhaustion (OE) 42.9% was seen amongst doctors and 57.1% had low degree of personal accomplishment (PA) compared to all other health care providers. Significant association was observed between two sub-scales of MBI (p<0.05). No significant association was observed in working hours, designated positions and burnout (p>0.05). Statistically weak correlation existed between burnout and patient safety culture (r=0.075, p=0.580). Awareness on incident reporting was in 43.3% of participants; of which 31% had reported at least one event in last 12 months. Overall, 76% employees consider their work unit reliable for providing safe patient care.

**Conclusions:**

Burnout was observed in employees, particularly high degree in attending physicians. However, team work, high level of personal accomplishment and incident reporting culture, served as protective factors for patient safety and safe working environment and culture.

## INTRODUCTION

Healthcare professionals are at an increased risk of psychological stress when their jobs are leaden with emotionally charged circumstances. This continuing emotional distress^1^ can lead to burnout syndrome, consequently leading to patient harm. This situation, a frequent topic of discussion, is a physiological response of an individual to long term stressors, which can be emotional and interpersonal.^2^ Burnout at workplace or professional burnout^3^ is a phenomenon, where employees at their jobs start to feel lack of energy, fatigue, being emotionally drained, mentally detached from their work/workplace and with pessimism seeping in.^4^,^5^

Professional burnout amongst healthcare providers is becoming common due to increased demand for patient centered care and administrative requisites compared to insufficient resources provided^3^. Burnout syndrome is associated with Emotional Exhaustion ‘EE’, Depersonalization ‘DP’, and Personal Accomplishment ‘PA’. In health care setting it is also associated with perceptions of patient safety culture and can compromise safe patient care.^6^

Comparative study was conducted amongst US Physicians with the objective to find changes in burnout levels and work-life balance satisfaction in 2011 relative to 2014. In 2011, 45.4% physicians experienced at least one symptom of burnout as compared to 2014 which was 54.4%. Work-life balance satisfaction was reduced from 48.5% in 2011 to 40.9% in 2014 (p<0.01).^7^ In December 2018, a cross-sectional study was conducted amongst 71 doctors of Ghulam Mohammad Mahar Teaching Hospital, Lahore Pakistan. Doctors completed abbreviated MBI questionnaire. Results showed moderate to high burnout i.e., 33.8%.^8^

Burnout can affect the job performance of healthcare workers, eventually leading to a decrease in the quality of care and alertness of healthcare professionals.^9^ Mental wellbeing and burnout can have multiple contributing factors such as increased patient demands, paperwork and dearth of support.^10^ Employees experiencing burnout have a greater tendency to distance themselves from job-associated colleagues, even their patients. Moreover, when discord is felt between the investment in their work and its return by healthcare providers, they become apprehensive regarding their efforts towards their jobs eventually developing a negative attitude even for their patients.^1^ This is known as the conservation of resources theory, which eventually results in a negative impact on patient safety practices. Patient safety is considered as one of the most important health issues by World Health Organization, and is defined as the absence of preventable harm and reduction in the risk of unnecessary harm associated with health care to an acceptable minimum for a patient.^11^ An estimated 134 million hospital-related adverse events occur every year in low and middle-income countries, even leading to an estimated 2.6 million deaths. These medication errors were found to be third in row after healthcare associated infections and safe surgery. During clinical judgment and medication administration, nurses who are fatigued are more prone to making errors. These errors are linked to adverse patient outcomes.^10^

Patient safety includes anticipation of error of diagnosis, medical error, patient identification**,** injury or other preventable harm to a patient during their hospital stay. Patient safety is not an easy target to achieve in healthcare settings, especially in developing countries due to resource constraints, shortage of well- trained qualified medical and allied health care professionals, lacking updated information technology, awareness or perception of patient safety.^12^ Studies highlight the importance of improving health systems as oppose to solely expecting individuals to reduce medical error.^13^

Providing a safety culture is undoubtedly an important aspect of patient care. It is the accumulative result of common behaviors, experiences, beliefs and values which reflect how things should be done in an environment. The first step in any organization to develop the culture of patient safety is to evaluate the current culture and then to identify the areas which require improvement.^9^ A cross-sectional study was conducted to assess association between patient safety culture and burnout in a pediatric hospital. A total of 148 professionals from three different hospitals were enrolled. It concluded that 44.6% of pediatric professionals were feeling burnout.^9^ Having an environment of patient safety culture improves the daily healthcare services as well as the overall climate of the organization. This in turn decreases the chances of physical and mental illness in healthcare providers.

Our study assessed the level of burnout amongst the health care workers who are directly involved in patient care and to establish whether there is a relationship between burnout and patient safety culture. At Manawan Hospital IHHN, Lahore patient safety has been one of our prime goals for the last five years and we have been adherently inculcating this culture. To ensure the development of this culture we need to ensure that our HCP are protected from the effects of burnout. This study will help understand prevalence if any, of burnout and recommend strategies to reduce burnout to ensure safe patient practices.

## METHODS

This cross-sectional study was carried out among health care providers directly involved in patient dealing/handling for a minimum of one year at Tehsil Head Quarter Manawan, a secondary care hospital, of Indus Hospital. Fifty-nine participants were included through an online survey using a non-probability consecutive sampling technique. Participants who gave their consent were able to access the questionnaire and fill it.

### Inclusion & Exclusion criteria

Employees of Manawan hospital with minimum work duration of one year were included while those with work duration of less than one year were excluded.

### Ethical approval

The study was approved by Institutional Research Board, IHHN, Ref.: IHHN_IRB_2022_12_010.

### Questionnaires

The survey Form consisted of two questionnaires, The Maslach Burnout Inventory (MBI)^14^ and Agency for Healthcare Research and Quality (AHRQ) Patient Safety Culture Survey;^15^ both translated in English and Urdu language. This self-administered survey on Redcap was conducted online, via email and WhatsApp.

Data was collected from April 01^st^ till 30^th^, 2023. Participants fulfilling inclusion criteria were approached. Level of burnout was assessed using a Maslach burnout inventory with 22 items grouped in three areas of Emotional Exhaustion, defined as emotionally drained due to work related stress, Depersonalization ‘DP’, as unable to connect with people on job, and Personal accomplishment ‘PA’ when one does not believe that they are good at what they do.^1^

To assess the patient safety culture of the hospital staff, AHRQ questions were divided into four parts: Patient safety perception, patient safety practices, event reports and near miss frequency.

### Statistical analysis

Sample size was determined using PASS 15 software, with an assumption of zero baseline correlation, a confidence level of 95%, and a power of 95%. We employed an alternative correlation value (p1) of 0.51 for emotional exhaustion and 0.45 for depersonalization as per AHRQ guidelines.^16^

Data was coded and analyzed using SPSS version 26. Quantitative variables such as age, year of experience, occupational exhaustion score, depersonalization score, personal accomplishment score and AHRQ subscale score was reported as mean ± SD. However, data of non-normal median (IQR) was reported. For qualitative variables such as staff position in hospital, working area, gender, level of occupational exhaustion, depersonalization and personal accomplishment assessment chi square was reported. Relationship between sub scales of MBI and AHRQ was done using spearmen correlation.

## RESULTS

The study involved participants from various working units within the organization. Specifically, it included 21 doctors, comprising consultants and medical officers, 20 nursing staff members, and six administrative staff members, primarily responsible for patient reception. Among the 59 participants, consisting of 48 females and 11 males, the median age was reported as 27 years (IQR=7) due to the non-normal distribution of data. The median work duration was two years. Table-I

**Table-I:** Characteristics of the study participants.

Median Age		27 years (IQR=7)
Gender	Male	11
	Female	48
Median Work duration		2 years
Percentage of staff from each department	Doctors	35.6%
	Nursing staff	33.9%
	Allied HealthCare staff	20.3%
	Administrative staff	10.2%

### Burnout among Participants

The study revealed a significantly higher level of emotional exhaustion among female participants (93.3%) compared to male participants (6.7%). Female participants also showed a consistent pattern of higher depersonalization (27.7%) without statistical significance (p>0.05). However, 57.4% of female participants reported low levels of personal accomplishment, Table-II.

**Table-II:** Level of Burnout among Genders.

Level of Occupational Exhaustion				
	Low degree	Moderate degree	High degree	P-value
***Gender***				
Female	19 (65.5%)	14 (93.3%)	14 (93.3%)	0.036
Male	10 (34.5%)	1 (6.7%)	1(6.7%)	
***Level of Depersonalization/ Loss of Empathy***				
Female	24 (51.1%)	10 (21.3%)	13 (27.7%)	0.308
Male	9 (75%)	2 (16.7%)	1 (8.3%)	
***Level of Personal Accomplishment***				
Female	27 (57.4%)	7 (14.9%)	13 (27.7%)	0.608
Male	5 (41.7%)	2 (16.7%)	5 (41.7%)	

Doctors experienced notably high levels of occupational exhaustion, with 14.3% reporting depersonalization compared to 25% among nursing staff. Personal accomplishment did not emerge as a significant factor affecting work attitudes, with 57.1% among doctors, 45% among nurses, and 75% among allied health workers (p>0.05). Notably, the study found no correlation between age and burnout but demonstrated a significant association between occupational exhaustion and depersonalization (p<0.05). A weak inverse correlation was observed between burnout and working hours, gender, and patient safety culture, suggesting that burnout was inversely related to the length of working hours (DP r= -0.51, p=0.702; EE r= -0.196, p=0.138). A very weak correlation was observed between depersonalization and patient safety perception among hospital staff (r= -1.25, p=0.354). Similarly, a very weak correlation was found between emotional exhaustion and patient safety culture (r= 0.075, p=0.580), with a very weak positive correlation with depersonalization (p=0.895) but a significant correlation with occupational exhaustion, Table-III.

**Table-III:** Level of Burnout among Healthcare Employees.

Level of Occupational Exhaustion					
	Doctors	Nursing Staff	Allied Healthcare Staff	Administrative Staff	P-value
Low degree	7 (33.3%)	13 (65%)	4 (33.3%)	5 (83.3%)	0.049
Moderate degree	5 (23.8%)	4 (20%)	6 (50%)	0	
High degree	9 (42.9%)	3 (15%)	2 (16.7%)	1 (16.7%)	
***Level of Depersonalization/ Loss of Empathy***					
Low degree	13 (61.9%)	11 (55%)	6 (50%)	3 (50%)	0.920
Moderate degree	5 (23.8%)	4 (20%)	2 (16.7%)	1 (16.7%)	
High degree	3 (14.3%)	5 (25%)	4 (33.3%)	2 (33.3%)	
***Level of Personal Accomplishment among healthcare workers***					
Low degree	12 (57.1%)	9 (45%)	9 (75%)	2 (33.3%)	0.346
Moderate degree	4 (19%)	2 (10%)	2 (16.7%)	1 (16.7%)	
High degree	5 (23.9%)	9 (45%)	1 (8.3%)	3 (50%)	

### Incident Reporting

The study examined near-miss frequency reports, revealing that 43.3% of the participants were aware of event reporting and practiced it. Clinical staff members accounted for 46.2% of near-miss reports, while paramedical and nursing staff reported a higher frequency of near misses and incidents (28.6%) compared to clinical and administrative staff (21.4% and 21.6%, respectively. Table-IV.

**Table-IV:** Questions to assess the Incident Reporting.

When a mistake is made, but is caught and corrected before affecting the patient, how often is this reported?					
	Never	Rarely	Sometimes	Most of the time	Always
Doctors	20%	40%	33.3%	46.2%	21.4%
Nursing Staff	40%	60%	22.2%	34.6%	28.6%
Allied Healthcare Staff	20%	0%	44.4%	11.5%	28.6%
Administrative Staff	20%	0%	0%	7.7%	21.4%
P value=0.455					
***When a mistake is made, but has no potential to harm the patient, how often is this reported?***					
Doctors	16.7%	40%	27.3%	52.9%	26.7%
Nursing Staff	33.3%	20%	45.5%	23.5%	46.7%
Allied Healthcare Staff	16.7%	30%	18.2%	17.6%	20%
Administrative Staff	33.3%	10%	9.1%	5.9%	6.7%
P value=0.760					
***When a mistake is made that could harm the patient, but does not, how often is this reported?***					
Doctors	0%	25%	36.4%	58.8%	27.8%
Nursing Staff	80%	37.5%	27.3%	23.5%	33.3%
Allied Healthcare Staff	20%	25%	18.2%	11.8%	27.8%
Administrative Staff	0%	12.5%	18.2%	5.9%	11.1%

P value= 0.527.

**Fig.1:**
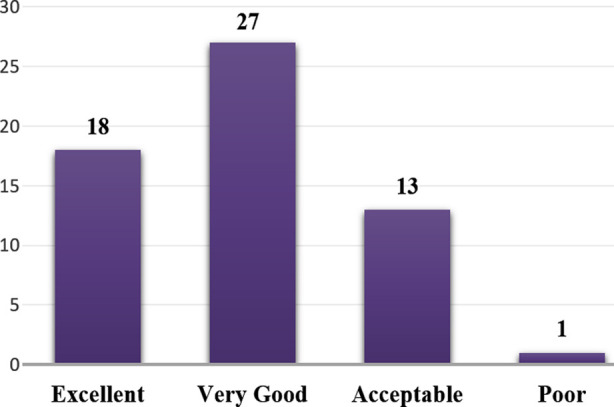
Overall Grade on Patient Safety of your work area/unit.

Approximately 76% of the participants considered their designated units safe for patients. However, the assessment of event reporting practices showed that 41.7% had never filled or submitted any event report in the past 12 months, while 31.7% had reported only one to two events. A smaller proportion reported three to four events (13.3%), six to ten events (5%), and 11-20 events (5%), with 1.7% reporting 11 or more events, Fig.1.

## DISCUSSION

Our study revealed significantly higher levels of emotional exhaustion among doctors (42.9%) compared to nursing and administrative staff. A study conducted on burnout among doctors and nurses also found higher emotional exhaustion levels among doctors (p=0.005). 17 Residents are also reported to have higher degree of burnout.^18^ Similarly, we found that an equal proportion of nurses experienced high levels of burnout and low personal accomplishment. A study on nurses who resigned from different U.S. hospitals identified burnout as a primary reason, which may be attributed to factors such as long working hours and a stressful environment.^19^ This included a negative working environment, increased workload and concerns about patient care.^20^

In our study, 75% of allied healthcare workers experienced low levels of personal accomplishment, possibly due to a lack of systematic workflow and inadequate human resources leading to increased workload. Administrative staff showed low levels of emotional exhaustion and depersonalization. Studies among pharmacists have also shown high burnout rates, based on increased emotional exhaustion and low personal accomplishment.^21^ A study reporting prevalence of professional burnout among the different healthcare professionals, rated from highest to lowest was 66% in nurses, 38.6% in physicians and 36.1% in administrative staff.^22^

Gender-wise, our findings indicated higher levels of burnout among female hospital employees compared to males. This could be attributed to various shift duties and low social support. A study conducted among general practitioners, dentists, and nurses concluded that females experience higher levels of emotional exhaustion leading to burnout.^23^,^24^

Contrary to earlier studies 25 we found no significant association between working hours and burnout among healthcare staff. A study from Oman concluded that there was an insignificant inverse relationship between workload (r= -0.056) and overall perceived patient safety (p>0.05).^12^

Incident reporting is an essential part of patient safety culture, also seen in various hospitals.^26^ According to our study, hospital staff is well aware of incident reporting. However, incident reporting practices are less common among doctors. It is more prevalent in nursing staff mainly due to the supervision or communication with their immediate supervisor/ manager. Doctors tend to report fewer incidents as it is time consuming to fill the form or maybe due to the length of the form. Lack of understanding of the form along with no feedback regarding the incident are contributing factors.^26^,^27^ Thus, the above finding point to the need to create an environment which supports physical and mental wellbeing to help to reduce professional burnout.

### Limitations

This study was conducted in one secondary care hospital in Pakistan, and its findings may not be generalized to the entire population of healthcare providers in the country. Additionally, the small sample size and the short study duration limit the study’s scope.

## CONCLUSION

The study revealed that employees in the hospital did not exhibit complete burnout, but there were significant levels of emotional exhaustion and depersonalization. However, these burnout levels did not significantly impact the patient safety culture. To improve patient safety and create a supportive work environment, contextual strategies based on employees’ burnout levels should be considered. Future studies should look deeper into the contributing factors leading to burnout.

### Authors` Contribution:

**AS** conceived, designed, did statistical analysis & is responsible for the integrity and accuracy of research.

**SZ** prepared draft and gave final approval for publication.

**SZ & AS** acquisition of data, manuscript writing, review and analysis.
